# Supplementary material for: Balancing noise and plasticity in eukaryotic gene expression
Source: BMC Genomics. 2012 Jul 27;13:343. doi: 10.1186/1471-2164-13-343 (PMC3539894; doi:10.1186/1471-2164-13-343)
Supplement: Additional file 1 — Supplement. Supplementary material including additional discussions on Taf1 and Tup1 effects and figures S1 to S16. [file 1471-2164-13-343-S1.pdf]

Supplement

Balancing noise and plasticity in gene expression

Djordje Bajić and Juan F. Poyatos

Logic of Genomic Systems Laboratory, Spanish National Biotechnology Centre, Consejo Superior de Investigaciones Científicas (CSIC), Madrid, Spain

TAF1 and regulation of gene expression variability

TBP-associated factor 1 (TAF1) is an essential component of the general transcription factor TFIID. It takes part of the eukaryotic transcriptional pre-initiation complex (PIC) that is involved in the transcription of ∼90% of yeast genes [1] (with the rest ∼10% controlled by SAGA). Genes associated to TFIID belong mostly to the LP and LNHP groups (while HNHP genes are enriched in stress-related functions and regulation by SAGA). In Fig. 2B, main text, we present evidence that LNHP genes –strongly plastic and histone-hyperacetylated genes regulated by TFIID– are more dependent on TAF1 than genes with low plasticity –which are neither plastic nor show high histone acetylation, but are also TFIID-dominated.

This suggests that when TAF1 (and TFIID) acts on LP genes it could merely constitute a structural component of the PIC, a component essential for transcription; in LNHP genes it gains importance also as a "*trans*–regulatory" component, in the sense of expression-level modulation. At these highly acetylated genes, regulation by TAF is likely to be achieved through its histone acetyltransferase activity (HAT) that was already related to gene expression variability [2]. On the other hand, HNHP genes are regulated by SAGA instead of TFIID, which also displays a HAT activity. Although the two HAT activities exhibited by SAGA and TFIID could be equivalent and functionally redundant [1], they are differentially involved in regulating the growth-stress genomic expression programs. As expected [3], this is also reflected in the noise-plasticity coupling.

Tup1 is an example of active maintenance of high nucleosome occupancy at promoters

What determines the presence of a stably positioned nucleosome at the promoter of some genes (in particular, HNHP genes)? Recent papers argued that high bendability of promoter DNA can partially account for these high occupancy levels [3–5]. However, it has also been observed that genes with high promoter occupancy tend to decrease it when activated [3]. This *change* in occupancy is not likely to be DNA encoded.

We found that chromatin regulators acting as repressors tend to regulate more strongly HNHP genes (Fig. 2, main text) and that the same proteins that upregulate most of LNHP genes tend to downregulate most of HNHP genes (Fig. 3, main text). Based on these findings we hypothesized that not only DNA properties but also mechanisms in *trans* help maintaining high occupancy levels at promoters. This view is supported by recent findings that showed how the co-repressor protein Tup1 acts stabilizing promoter nucleosomes at highly plastic genes, a strategy not identified before to our notice [6]. It could be interesting to explore in the future if this kind of mechanisms could be widely found as occupancy modulators.

References

1. Huisinga KL, Pugh BF (2004) A genome-wide housekeeping role for tfiid and a highly regulated stress-related role for saga in saccharomyces cerevisiae. Molecular Cell 13: 573 - 585.

2. Choi JK, Kim YJ (2008) Epigenetic regulation and the variability of gene expression. Nature genetics 40: 141–7.

3. Kim SC, Choi JK (2011) Controlling transcriptional programs for cellular adaptation by chromatin regulation. Molecular bioSystems 7: 1713–9.

4. Kaplan N, Moore IK, Fondufe-Mittendorf Y, Gossett AJ, Tillo D, et al. (2009) The DNA-encoded nucleosome organization of a eukaryotic genome. Nature 458: 362–6.

5. Field Y, Fondufe-Mittendorf Y, Moore IK, Mieczkowski P, Kaplan N, et al. (2009) Gene expression divergence in yeast is coupled to evolution of DNA-encoded nucleosome organization. Nature genetics 41: 438–45.

6. Rizzo JM, Mieczkowski Pa, Buck MJ (2011) Tup1 stabilizes promoter nucleosome positioning and occupancy at transcriptionally plastic genes. Nucleic acids research : 1–17.

7. Lehner B (2010) Conflict between Noise and Plasticity in Yeast. PLoS Genetics 6: e1001185.

8. Benjamini Y, Hochberg Y (1995) Controlling the false discovery rate: A practical and powerful approach to multiple testing. Journal of the Royal Statistical Society Series B (Methodological) 57: pp. 289-300.

9. Holstege FC, Jennings EG, Wyrick JJ, Lee TI, Hengartner CJ, et al. (1998) Dissecting the regulatory circuitry of a eukaryotic genome. Cell 95: 717–28.

10. Tirosh I, Wong KH, Barkai N, Struhl K (2011) Extensive divergence of yeast stress responses through transitions between induced and constitutive activation. Proceedings of the National Academy of Sciences of the United States of America 108: 16693–8.

11. Tirosh I, Barkai N (2008) Two strategies for gene regulation by promoter nucleosomes. Genome research 18: 1084–91.

12. Choi JK, Kim YJ (2009) Intrinsic variability of gene expression encoded in nucleosome positioning sequences. Nature genetics 41: 498–503.

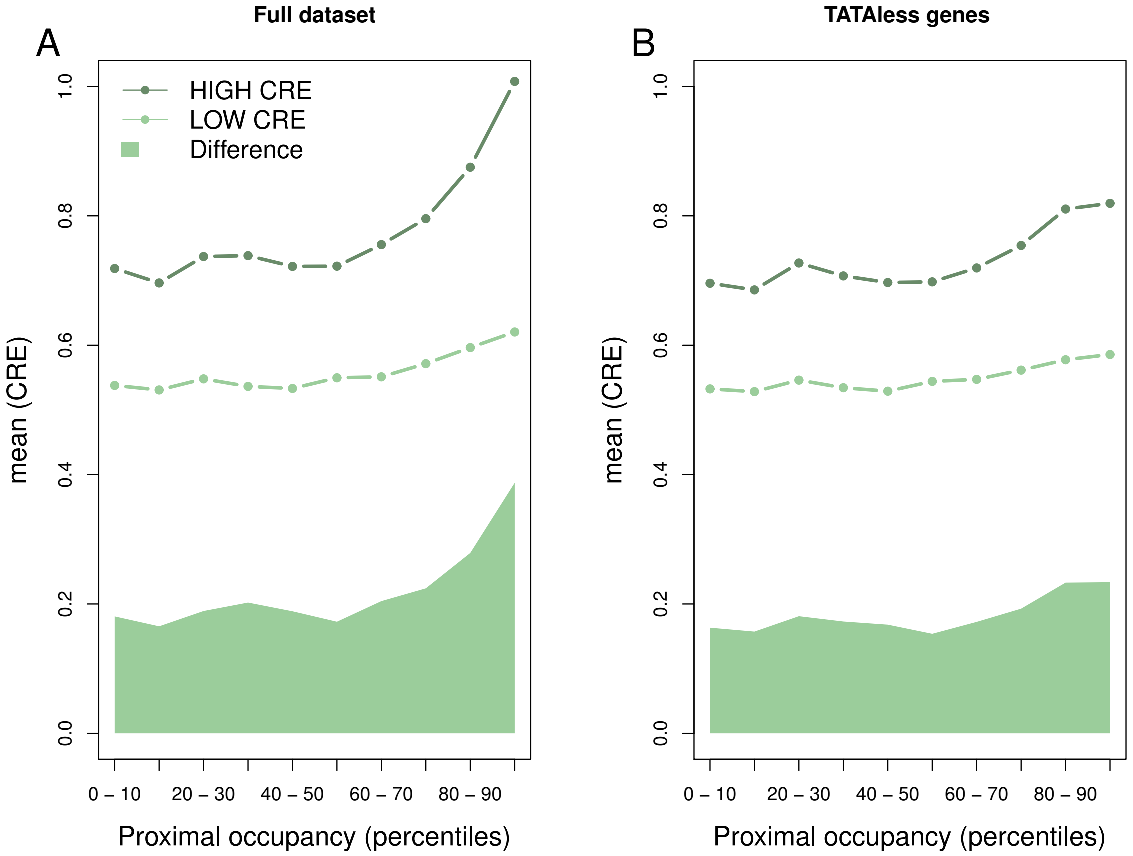

Figure S1. Chromatin regulation and proximal nucleosome occupancy. In Figure 1, main text, each proximal (nucleosome) occupancy bin was divided into HIGH CRE and LOW CRE subgroups, i.e., above or below the median CRE in each bin. This figure supplements Fig. 1 by showing the average CRE score for each of these bins (and the difference between them at the bottom, shaded in green).

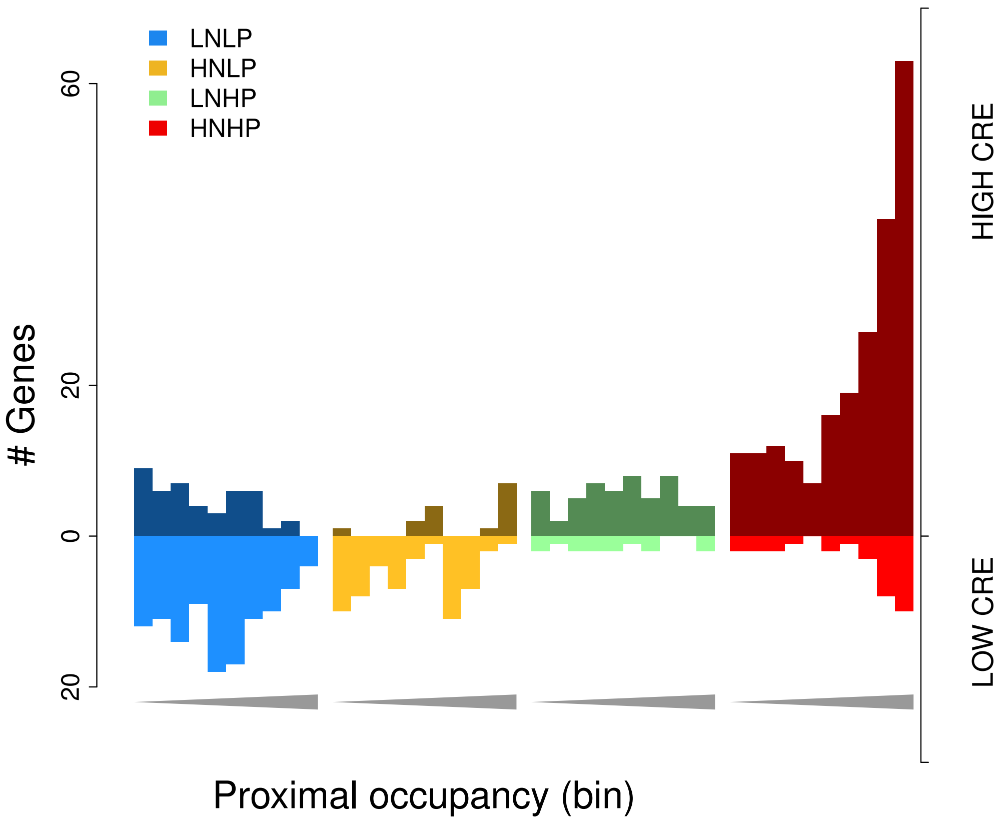

Figure S2. Distinctive noise-plasticity phenotypes show differential patterns of chromatin regulation and proximal nucleosome occupancy. We sorted genes (n=2045) in high and low CRE, i.e., above or below the median, and also in ten equally sized bins of proximal occupancy. We only selected those genes exhibiting extreme phenotypes in terms of noise and plasticity; e.g., high-noise high-plasticity (HNHP), low-noise low-plasticity (LNHP), etc (see Methods in main text). We can observe that the groups with high plasticity are enriched in genes belonging to the HIGH CRE subset. In addition, the HNHP group shows promoters highly occupied by nucleosomes.

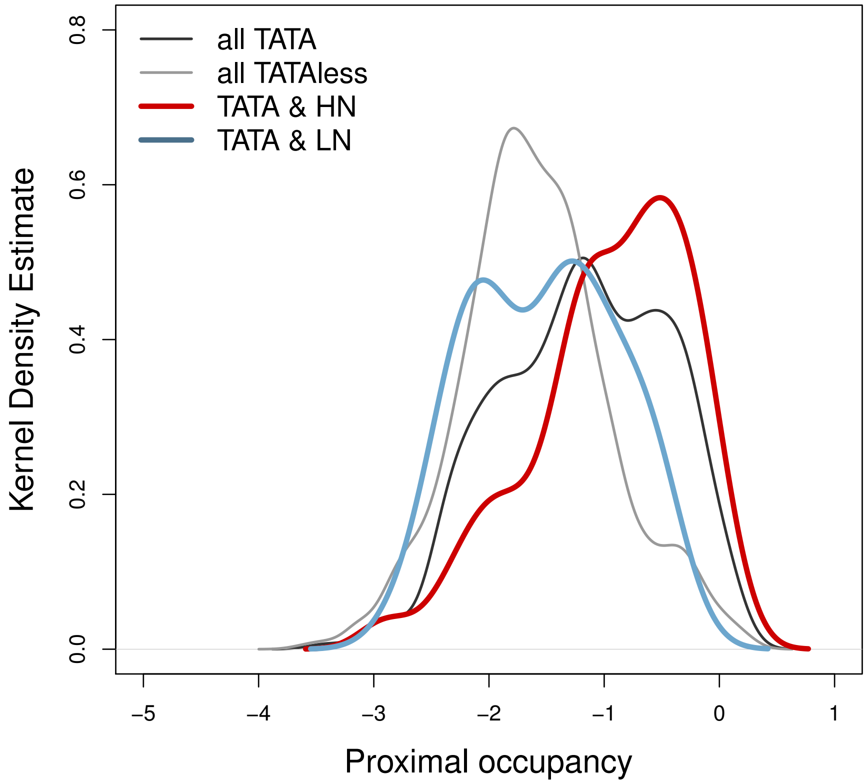

Figure S3. TATA boxes enhance noise only in highly occupied promoters. Here we plotted the distribution of proximal (nucleosome) occupancies observed in the TATA-containing subset of “high noise” genes (HN, noise above percentile 75 of full dataset) and “low noise” genes (LN, noise below percentile 25), as well as the distribution of the whole TATA and TATAless subgroups. Noisy TATA-containing genes show highly occupied promoters. In contrast, quiet TATA-containing genes display nucleosome depleted regions more similar to the TATAless group.

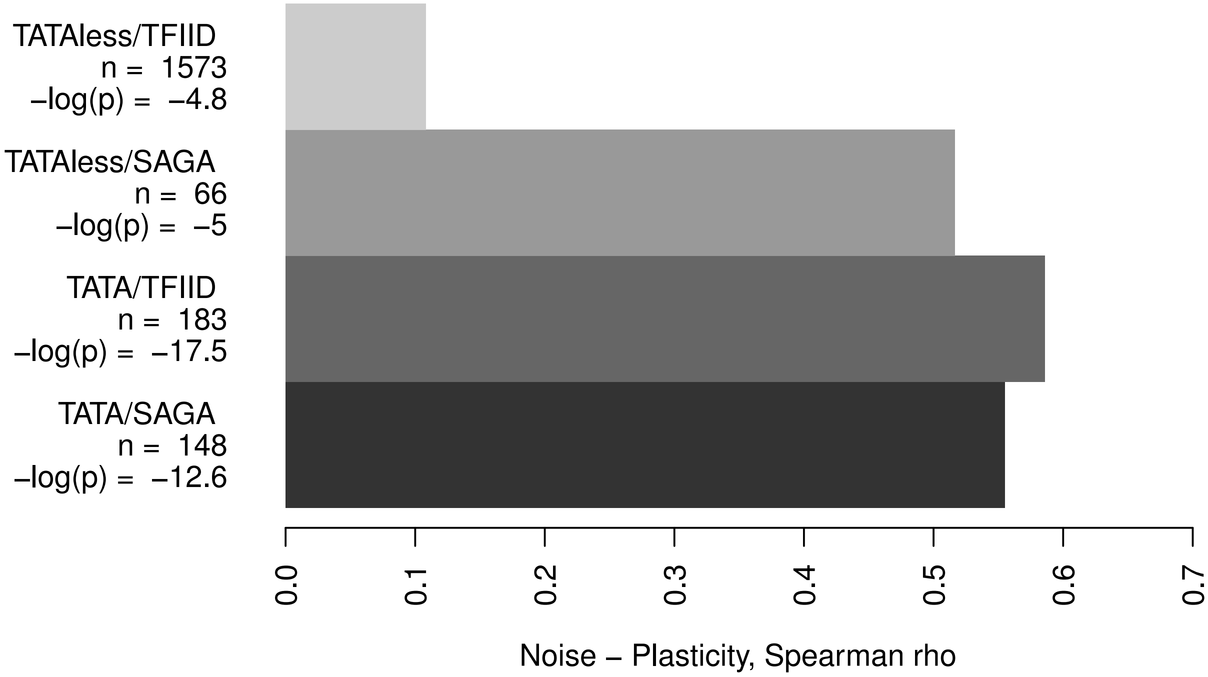

Figure S4. Bursting transcription relates to noise-plasticity coupling. We show the Spearman ρ correlation for TATA-containing or TATAless genes and SAGA-dominated or TFIID-dominated genes. Most of the SAGA-regulated genes are also TATA-containing [1], and it has been shown that TATA boxes strongly enhance noise-plasticity coupling [7]. However, a SAGA-dominated transcription can also enhance this coupling even in TATAless genes. This represents an additional evidence of the relation between coupling and transcription initiation.

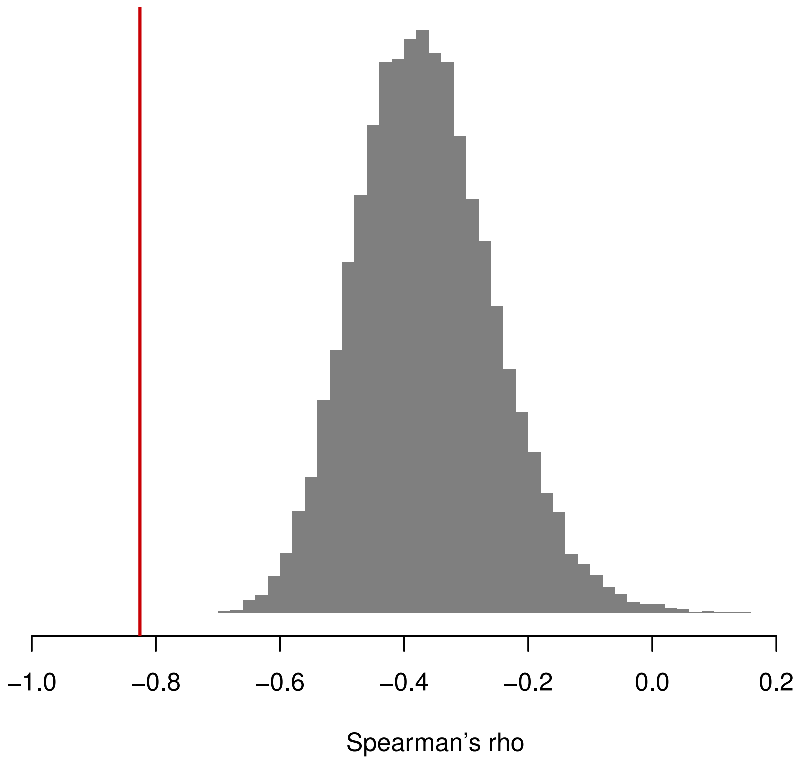

Figure S5. Baseline negative correlation between chromatin regulators over HNHP and LNHP groups. We report in figure 2 (main text) a negative correlation ($\rho =$ -0.82) between the regulation strength of different chromatin regulators over HNHP and LNHP groups normalized by its strength over the entire HP group. This ratio introduces a baseline (negative) correlation. To demonstrate that the observed correlation is nevertheless significant, we computed its null distribution by a permutation test (within the 510 genes of the HP group, we selected random groups of genes with the sizes of the HNHP and LNHP subgroups 10000 times and scored the corresponding correlation). In this figure, we plot the distribution of correlation coefficients (mean$=-0.36$, maximal random negative correlation $= -0.69$). The observed value (-0.82, red line) is therefore clearly significant.

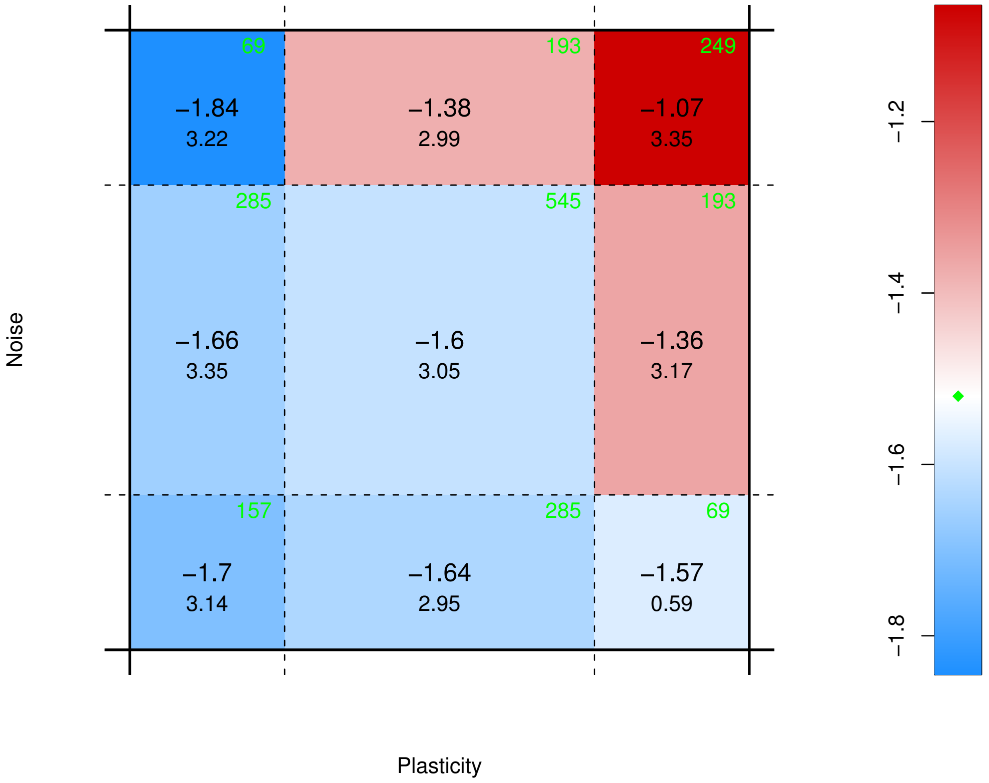

Figure S6. Proximal nucleosome occupancy distribution as a function of noise and plasticity level. Each region of the square is colored according to the mean proximal nucleosome occupancy of the genes located in it (number of components n for each box shown in green in the upper-right corner). Bounds represent percentiles 25 and 75 in both noise and plasticity, so that the four corners correspond to the four classes LNLP, HNLP, etc. The green dot in the colorbar represents the whole-dataset mean. In addition to the coloring, the mean value of the proximal nucleosome occupancy (as $\log_2$ ratio, see Methods) in each square is shown in the center of each square. Below this number, we show the $-\log_{10}(P)$; these P-values were obtained by bootstrapping (10000 times) and corrected for multiple testing [8]. It can be seen that covered promoters, i.e. with the highest occupancy levels, concentrate in the high-noise high-plasticity (HNHP) corner.

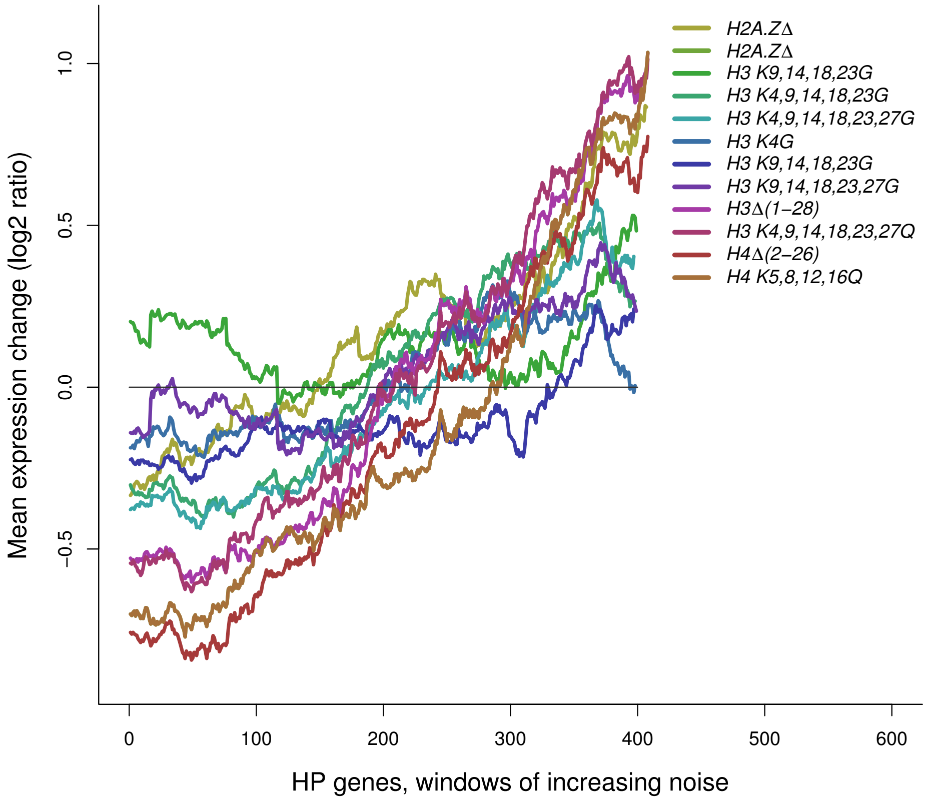

Figure S7. Histones differentially regulate HNHP and LNHP genes. We ordered the HP group ($n = 511$) by ascending noise and performed a sliding window analysis (window size $= 100$ genes) of the expression values after different histone perturbations (this relates to the orange dots shown in Fig. 3, main text; note that the two curves describing perturbations of H2A.ZΔ correspond to two different experiments, see Methods in main text). Genes whose plasticity is not coupled to noise tend to reduce their expression level; therefore, histones act favoring their expression. On the contrary, the same perturbations tend to increase the expression of genes with high noise, indicating a repressive action.

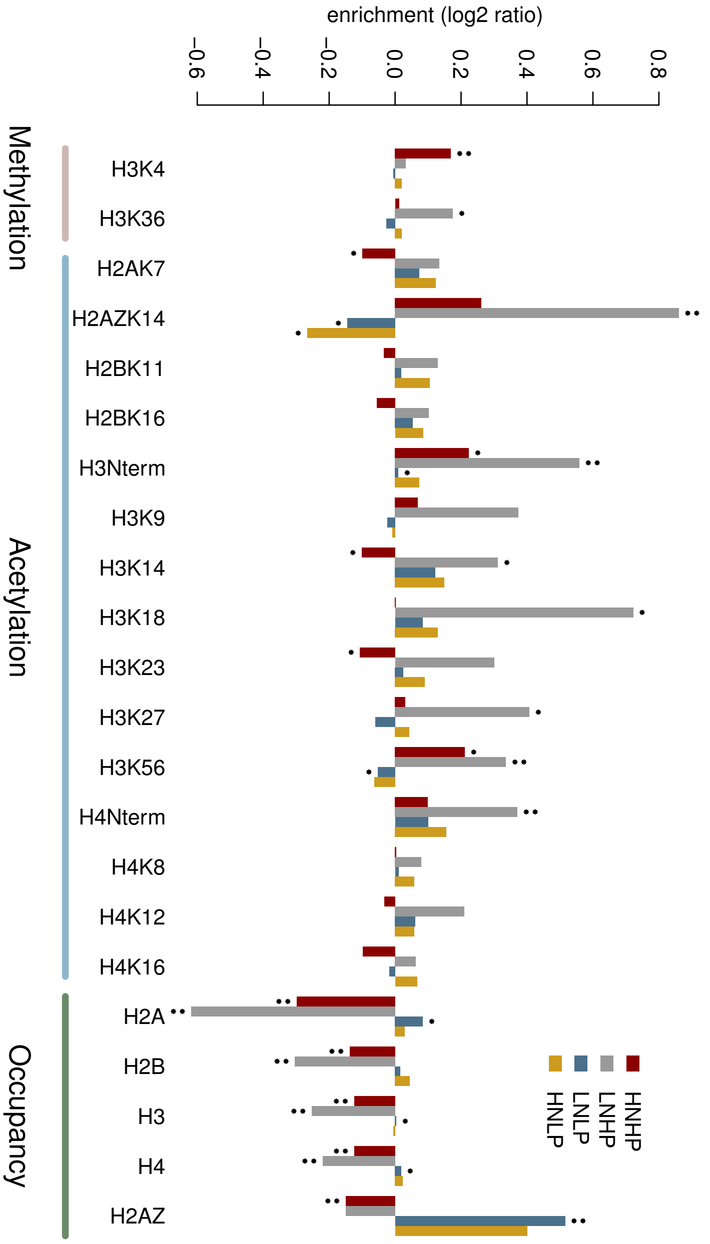

Figure S8. Histone modifications in noise-plasticity classes. We show the enrichment in different histone modifications at promoters, using nucleosome-normalized data. We used ChromatinDB (*http://www.bioinformatics2.wsu.edu/cgi-bin/ChromatinDB)*) as data source. At each bar, one asterisk is shown if enrichment is significant at $< 10^{-2}$ level and two asterisks if it is significant at $< 10^{-4}$. These P-values were corrected for multiple testing [8].

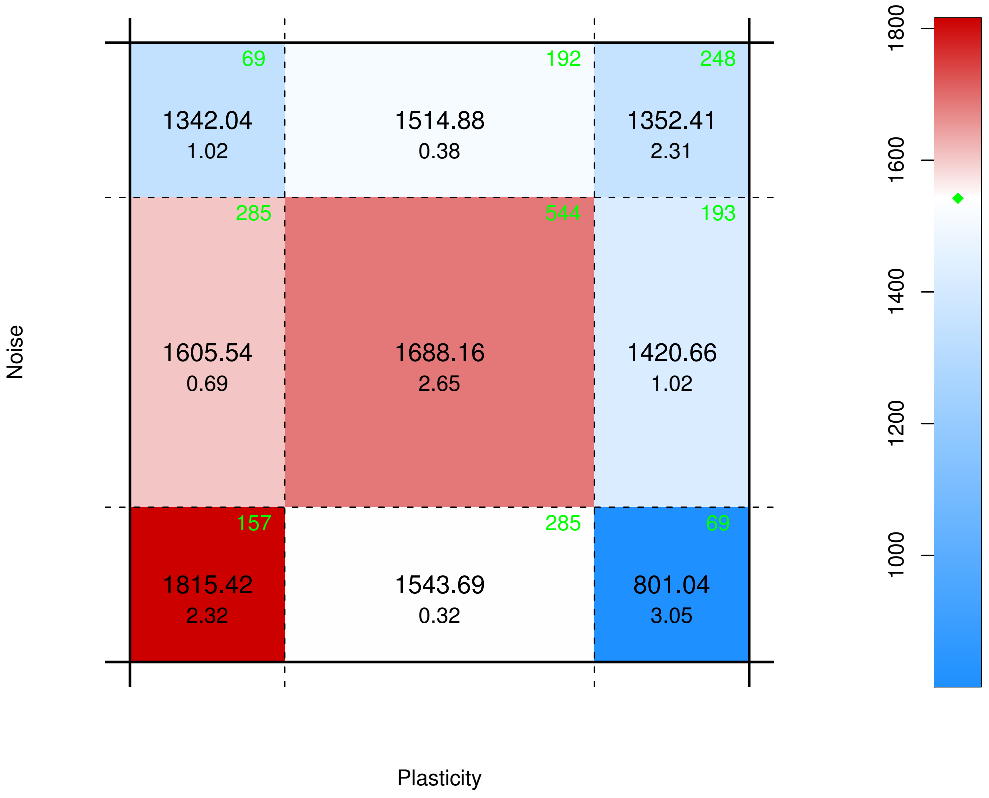

Figure S9. ORF length as a function of noise and plasticity levels. The distribution of ORF lengths is depicted in a similar way as Fig. S6. We observe that noise is negatively correlated with ORF length in the low plasticity region, probably due to its influence on translation efficiency. In the high plasticity region, we can see that the LNHP group is enriched in short ORFs. This is associated to the small size of ribosomal protein genes, the dominant functional class in this group. See main text for details.

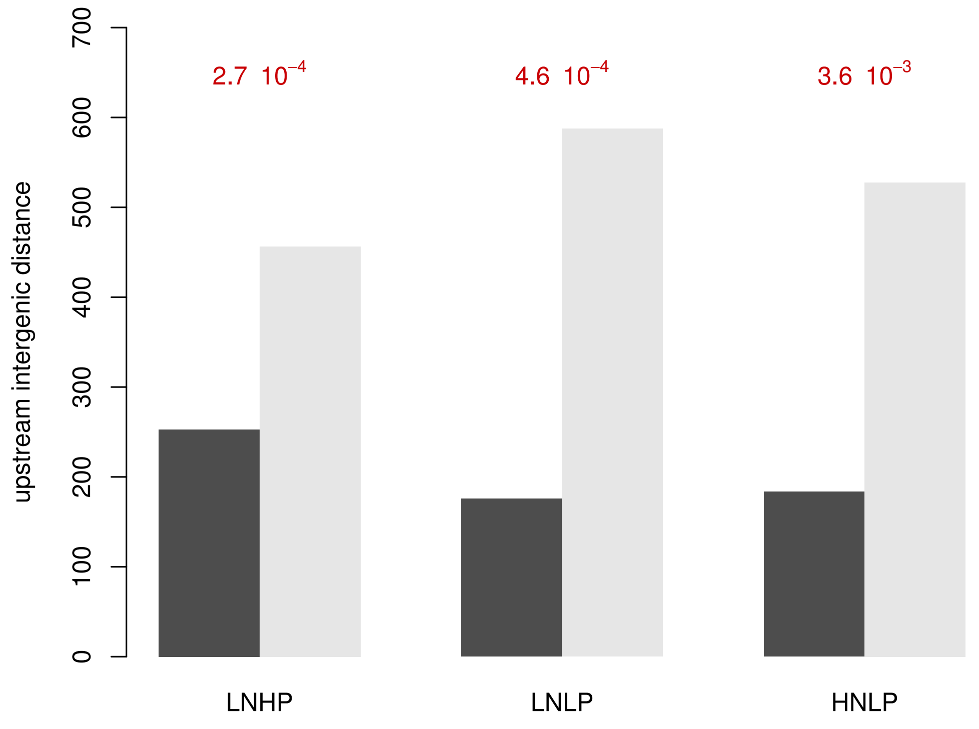

Figure S10. Bipromoters in the LNHP, LNLP and HNLP are associated to short intergenic distances. We plot the upstream intergenic distances for those genes within each class that presents (dark gray) or does not present (light gray) bipromoters. For each class, bipromoters exhibit smaller intergenic distances, significance given by Wilcoxon tests)

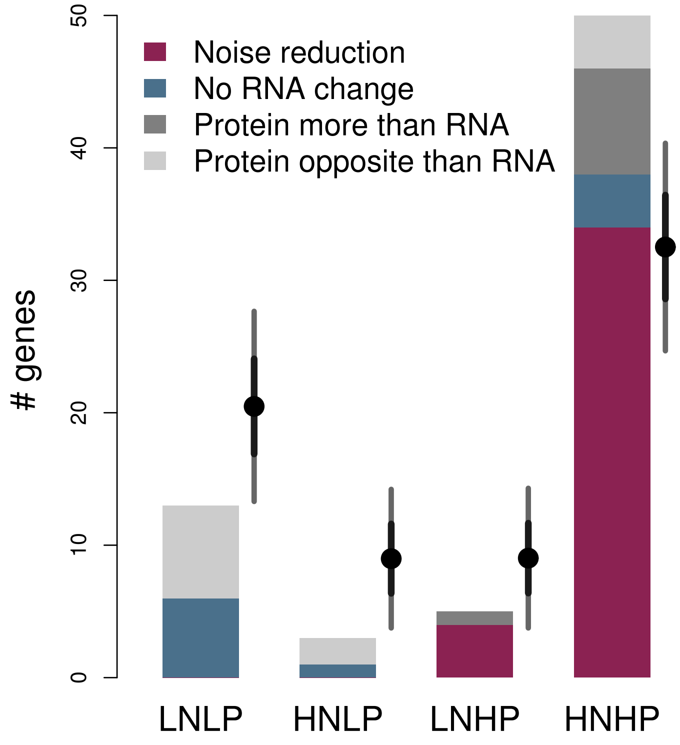

Figure S11. Post-transcriptional regulation is enriched in HNHP group. We present the number of genes in each group that is subjected to post-transcriptional regulation (PTR) under osmolarity stress conditions. Blades depicted next to each bar represent the mean ± two standard deviations obtained with a nonparametric permutation test. HNHP genes are enriched in PTR, while groups with low plasticity are less regulated than expected at random. We also showed within each bar the different modes of PTR identified. Noise-reducing PTR was only observed in highly plastic genes. The few genes with low plasticity that are post-transcriptionally regulated, showed changes in protein abundance despite no change in RNA, or even opposite changes. This suggests that the plasticity of these genes could be higher than detectable by variations in mRNA.

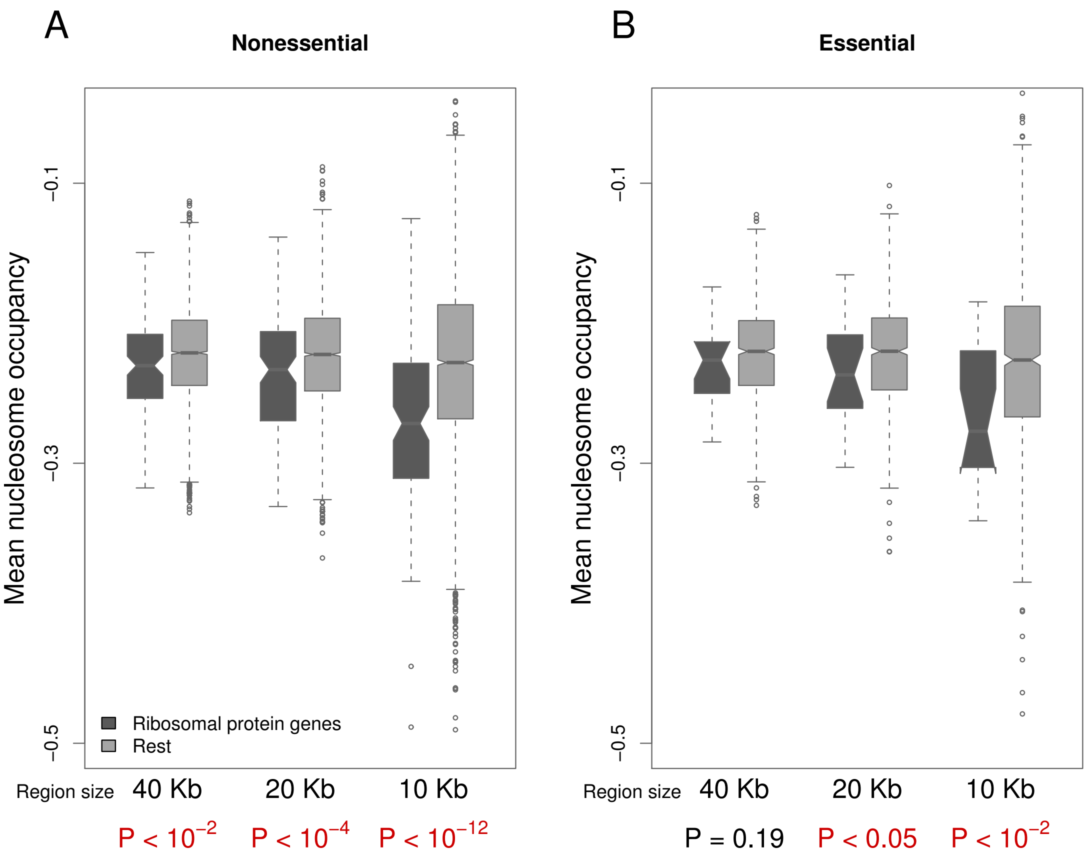

Figure S12. Ribosomal genes are located in broad open-chromatin genomic regions. We evaluated if ribosomal protein genes are located in broad regions of open chromatin. Taking each ribosomal protein gene TSS as the focal point, we measured the mean nucleosome occupancy in 5, 10 and 20kb upstream and downstream (making up regions of 10, 20 and 40kb, as shown in the figure labels). Ribosomal genes tend to be located in regions with significantly lower occupancy than non-ribosomal genes. Since essential genes are expected to be in low occupancy regions (exhibiting in consequence low noise) regions, we analyzed separately essential and nonessential genes, observing the same pattern.

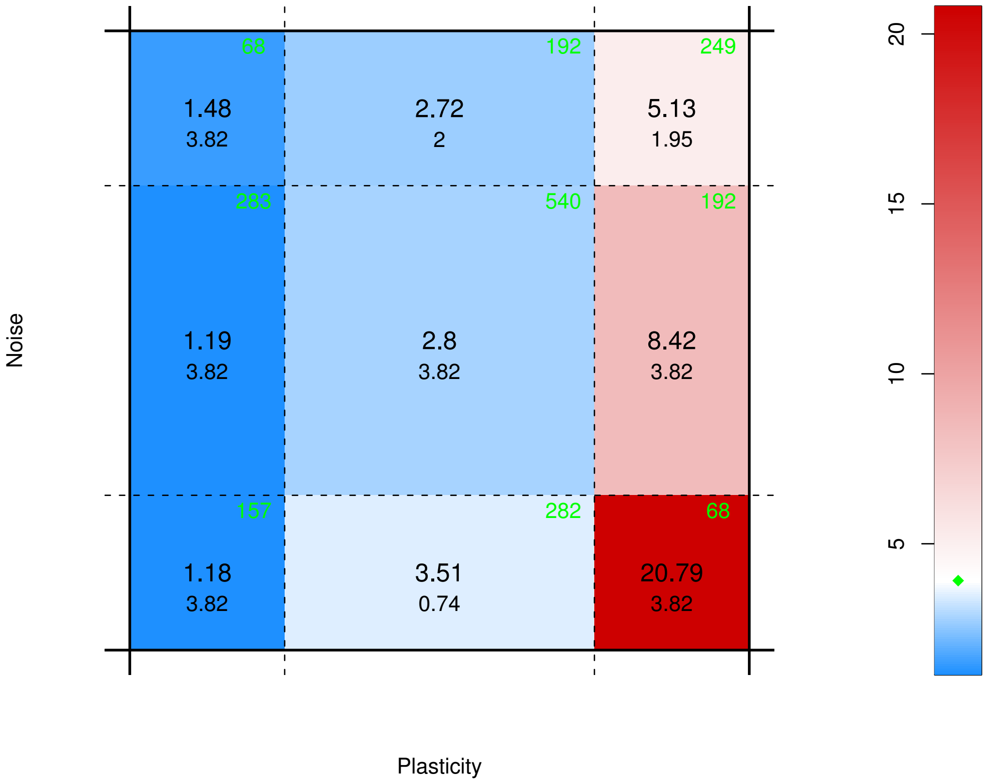

Figure S13. Expression level as a function of noise and plasticity levels. HP genes present high mRNA expression levels (mRNA copies/cell [9]). The highest average expression level within HP is found in the LNHP class that is highly enriched in ribosomal protein genes (known to be highly expressed). Interestingly, HP genes show higher expression level than LP genes even when the expression data considered was produced in rich medium (in which stress-response genes, enriched in HNHP, are repressed). This suggests that repression by nucleosomes that hinder transcription could be “leaky” with high basal expression level. In addition, this might reflect the type of gene expression evolution of these genes (see [10]).

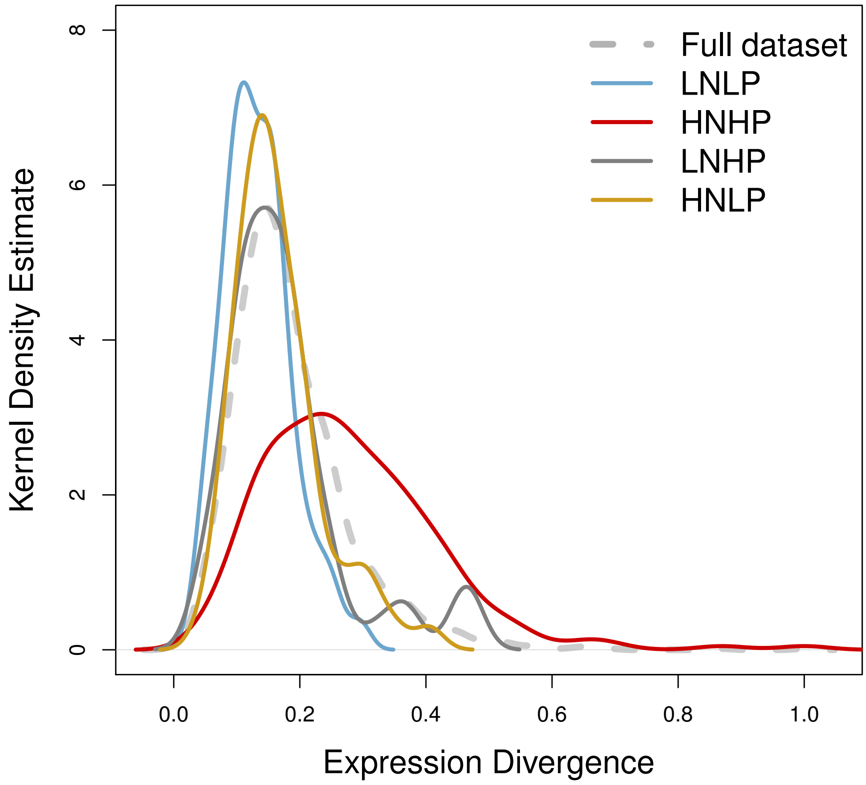

Figure S14. Gene expression divergence is coupled to noise and plasticity with the same pattern. Although HNLP and LNHP genes exhibit higher average expression divergence than LNLP genes, it seems that expression divergence is also strongly coupled to both noise and plasticity as previously suggested [11, 12].

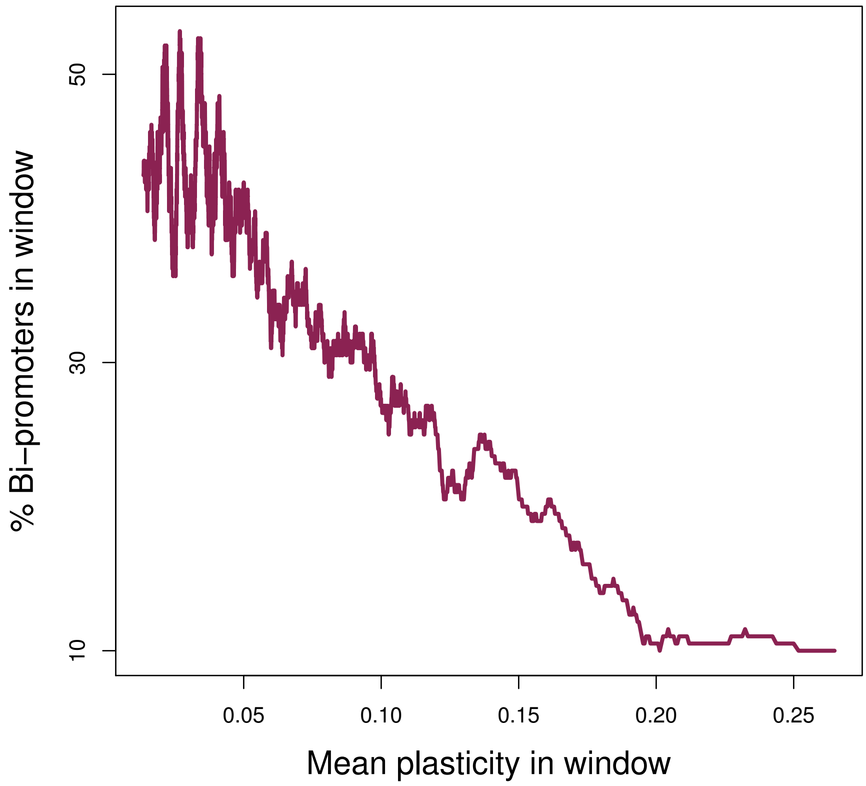

Figure S15. Bipromoter frequency strongly covariates with plasticity. We ordered genes in ascending plasticity ($n = 5111$) and then performed a sliding window analysis using a window size of 200 genes. For each window, we plot the percentage of genes transcribing from a bipromoter against the average plasticity in the window. The percentage of bipromoters decreases from ~50% (lowest plasticity values) to 10% (highest plasticity regime).

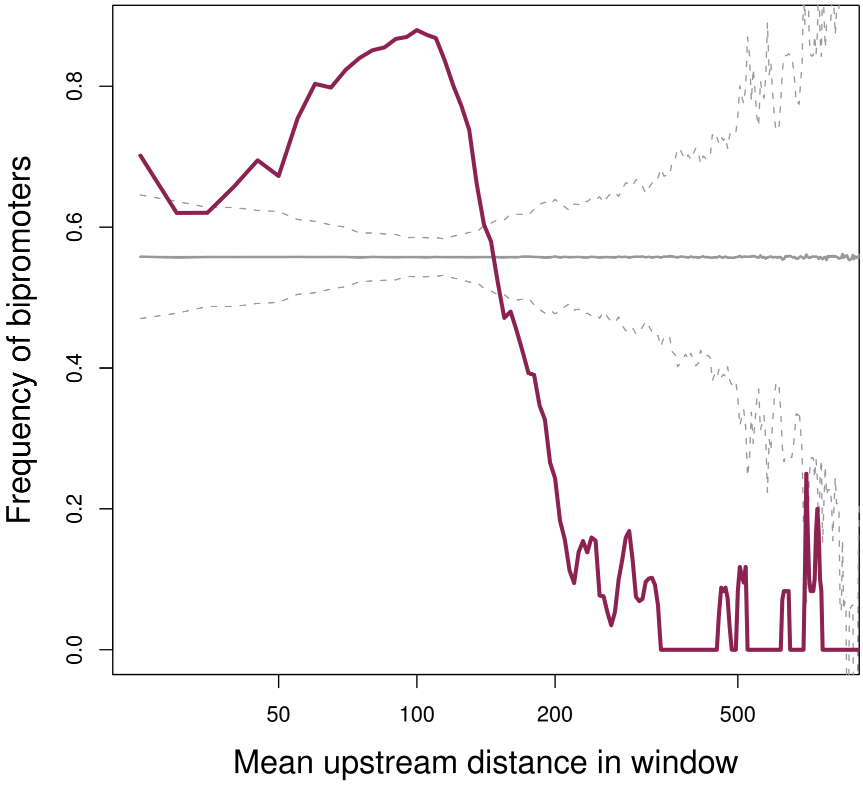

Figure S16. Intergenic distance predicts presence of bipromoters. We computed the frequency of genes starting at bipromoters considering a given intergenic distance by means of a sliding windows analysis (window size of 50bp, step-size of 10bp; note that the number of genes observed for each window is not fixed) using $n = 4856$ ORFs (including noncoding) with divergently oriented upstream partners. To estimate the null behavior, we conducted a permutation test by randomizing genes within each window (10000 times). We then computed the mean frequency of bipromoters randomly found (solid line) $\pm$ two standard deviations (dashed lines). As expected, bidirectional promoters are greatly enriched at low intergene distances, peak $\sim$ 100bp. At distances approximately bigger than 150bp, which is approximately the distance potentially covered by a nucleosome or a NDR, the frequency of bipromoter decreases. This strongly indicates that distance constraints are globally the most important in determining the presence of bipromoters.
